# Uncovering the co-evolutionary network among prokaryotic genes

**DOI:** 10.1093/bioinformatics/bts396

**Published:** 2012-09-03

**Authors:** Ofir Cohen, Haim Ashkenazy, David Burstein, Tal Pupko

**Affiliations:** Department of Cell Research and Immunology, George S. Wise Faculty of Life Sciences, Tel Aviv University, Tel Aviv 69978, Israel

## Abstract

**Motivation:** Correlated events of gains and losses enable inference of co-evolution relations. The reconstruction of the co-evolutionary interactions network in prokaryotic species may elucidate functional associations among genes.

**Results:** We developed a novel probabilistic methodology for the detection of co-evolutionary interactions between pairs of genes. Using this method we inferred the co-evolutionary network among 4593 Clusters of Orthologous Genes (COGs). The number of co-evolutionary interactions substantially differed among COGs. Over 40% were found to co-evolve with at least one partner. We partitioned the network of co-evolutionary relations into clusters and uncovered multiple modular assemblies of genes with clearly defined functions. Finally, we measured the extent to which co-evolutionary relations coincide with other cellular relations such as genomic proximity, gene fusion propensity, co-expression, protein–protein interactions and metabolic connections. Our results show that co-evolutionary relations only partially overlap with these other types of networks. Our results suggest that the inferred co-evolutionary network in prokaryotes is highly informative towards revealing functional relations among genes, often showing signals that cannot be extracted from other network types.

**Availability and implementation:** Available under GPL license as open source.

**Contact:** talp@post.tau.ac.il.

**Supplementary information:** Supplementary data are available at *Bioinformatics* online.

## 1 INTRODUCTION

Prokaryotic genomes are highly variable in their size (Koonin and Wolf, 2008; Mira *et al*., 2002). Comparative genomic analyses revealed that variability in gene content among genomes is a major factor contributing to this size variability (Konstantinidis and Tiedje, 2004; Pal *et al*., 2005). This substantial variability is mainly the result of gene acquisition via Horizontal Gene Transfer (HGT) (Gogarten and Townsend, 2005) and gene loss, e.g. as a result of reductive evolution (Moran, 2003). Gene content across genomes is compactly represented by phyletic patterns (also known as phylogenetic profiles), in which the presence or absence of each COG (Clusters of Orthologous Genes) in each genome is represented as a 0/1 binary character (see Cohen *et al*., 2008 for details).

Genes can have correlated evolutionary histories. This may reflect mutual dependency constraints, e.g. when these genes correspond to proteins that are part of a complex. Detecting such co-evolutionary interactions is important for understanding genome evolution as a coordinated process rather than as a collection of single evolutionary descriptions of each gene.

For protein and RNA evolutionary studies, a great deal of effort was invested in developing tools that can detect co-evolving sites (e.g. Ashkenazy and Kliger, 2010; Valencia and Pazos, 2002), the most accurate of these explicitly account for the phylogenetic tree that generated these sequences (Dutheil *et al*., 2005; Pollock *et al*., 1999; Poon *et al*., 2007). These approaches, in essence, search for correlated evolutionary events among sites, i.e. at two co-evolving positions, the substitutions occur in a pattern that is different from that expected by chance for two independently evolving sites. A similar approach can be used for the detection of co-evolving genes, in which gains and losses tend to co-occur in the same lineages. Accordingly, perfectly correlated genes are co-gained and co-lost during their entire history.

Methods to search for co-evolving genes were previously developed (Dutkowski and Tiuryn, 2009; Ettema *et al*., 2001; Glazko and Mushegian, 2004; Huynen and Snel, 2000; Marcotte *et al*., 2000; Pellegrini *et al*., 1999; Wu *et al*., 2003; Zheng *et al*., 2002; Zhou *et al*., 2006). Phylogeny-based examples include methods that employ the maximum parsimony framework (e.g. Campillos *et al*., 2006; Cordero *et al*., 2008) and methods that rely on explicit evolutionary models of co-evolution (e.g. Barker *et al*., 2007). However, using the maximum parsimony criterion may be misleading, in particular when there is substantial variability in branch lengths (Felsenstein, 1978; Pol and Siddall, 2001; Swofford *et al*., 2001; Yang, 1996). Furthermore, we have previously shown that maximum parsimony is less accurate for the inference of gain and lost events compared with model-based approaches (Cohen and Pupko, 2011). Ideally, methods that rely on models that explicitly take into account co-evolution among genes, rather than methods that only use models to test for deviation from the independence assumption should preferably be used. However, explicit modeling of co-evolutionary interactions is currently too computationally extensive to allow the analysis of large datasets, such as those studied in this work.

Here we present a novel probabilistic methodology to detect co-evolutionary interactions from phyletic patterns. We apply our methodology to analyze hundreds of genomes and thousands of COGs and provide novel insights into the co-evolutionary dynamics of genes across the bacterial domain.

## 2 METHODS

### 2.1 Evolutionary model and mapping branch-specific events

The input for our methodology includes the phyletic pattern (presence/absence profile) of COGs and the species tree. The phyletic pattern was extracted from eggNOG (Muller *et al*., 2010), an extended version of the COG database (Tatusov *et al*., 1997). Notably, across the manuscript, we often use term gene as a synonym for the term COG, to improve the readability of the text. The analysis is performed on an extensive dataset of 282 prokaryotes. A comprehensive pre-computed microbial species tree was used as input (Dehal *et al*., 2010; Price *et al*., 2010). Phyletic pattern data and the input tree are provided as Supplementary Files S1 and S2.

The gain and loss dynamics are modeled as a stationary continuous-time Markov process that allows variability among protein families for both gain and loss rates (Cohen and Pupko, 2010). The model's free parameters are unknown and are estimated numerically based on the data using the maximum likelihood criterion. Since branch lengths of the input tree are given in units of substitutions per site, we also estimate a branch-lengths-scaling parameter, which acts to transform all branch lengths to unit of gain and loss events.

Given the evolutionary model, for each COG and for each tree branch, the expected numbers of gain and loss events were inferred using the stochastic mapping methodology (Cohen and Pupko, 2010; Minin and Suchard, 2008; Nielsen, 2002).

### 2.2 Simulations-based computation of co-evolution

Let *n* denote the number of species. Thus, there are 2*n*−2 branches in our rooted phylogenetic tree. For a specific COG, the above mapping of events to each branch of the tree can be represented by a vector of size 4*n*−4, where half of the entries are used to represent gain events, and the remaining ones represent loss events. We next compute the correlation between the evolutionary histories of a pair of COGs by computing the Pearson's correlation between the two 4*n*−4 dimensional vectors.

The correlation coefficient depends on the number of gain and loss events along the tree. Two COGs that are present in all analyzed species are perfectly correlated; however, this does not necessarily reflect co-evolution. To this end, we only search for co-evolving COGs that experienced a minimal number of gain and loss events across the phylogeny (see also Dutheil *et al*., 2005). Specifically, we compute for each COG a value, which we term exchangeability, that is the average of the posterior expectation of gain events and loss events across the tree. The distribution of the Pearson's correlation coefficients was found to highly depend on the minimal exchangeability for independently simulated genes. Pairs with low exchangeability may show extremely high correlations by chance (Supplementary Fig. S1A). We thus tested for co-evolution only in pairs of COGs with exchangeability above a certain threshold, for both COGs. Too high a threshold may lead to filtering true co-evolving pairs. Too low a threshold would lead to exceedingly high number of pairs tested, which in turn may reduce the ability to detect true co-evolving pairs due to multiple testing. We set the exchangeability threshold to five events, to balance between these two considerations.

Positive correlations between two COGs do not necessarily indicate genuine co-evolution, e.g. because high number of events are expected in longer branches for both COGs and vice versa for shorter branches. To test whether an observed correlation value for a given pair is statistically higher than that expected for two independently evolving COGs, we associate each observed correlation coefficient between a pair of COGs with a *P*-value for which the null distribution of the correlation coefficients was computed using parametric bootstrap as follows: for each pair of COGs, we computed the minimum value of exchangeability between them. The observed correlation coefficient for this pair was then compared with a null distribution of independently evolving pairs with comparable minimal exchangeability values, to generate a *P*-value. As stated above, the distribution of correlation coefficients depends on the minimal exchangeability, and thus, the entire range of exchangeability was partitioned into bins, each representing a different null distribution. The bins used are provided in Supplementary Figure S1.

For the size of data analyzed in this study, millions of possible pairs are statistically tested for co-evolution, which necessitates controlling the False Discovery Rate (FDR; Benjamini and Hochberg, 1995). An FDR value of 0.01 was chosen, which let us to consider pairs with *P*-value lower than 1.38E-05.

### 2.3 Analysis of the co-evolutionary network

The network of co-evolving COGs was analyzed and visualized using Cytoscape (Smoot *et al*., 2011) and Pajek (Batagelj and Mrvar, 2002). The clustering of the network was conducted Transitivity Clustering algorithm (Wittkop *et al.*, 2011) with the default parameters.

### 2.4 Comparing co-evolution network with other biological networks

The protein–protein interactions (PPI), genomic proximity, co-expression and gene fusion networks were extracted from STRING 9.0 (Szklarczyk *et al*., 2011). For each type of biological association, the network was reconstructed by considering reliable connections between COGs with score of at least 700 (‘high confidence’). The metabolic network was extracted from KEGG by considering metabolic connections between COGs that take part in the same metabolic pathway (Kanehisa *et al*., 2012).

## 3 RESULTS

### 3.1 Reconstruction of the co-evolutionary network

**Fig. 1.**
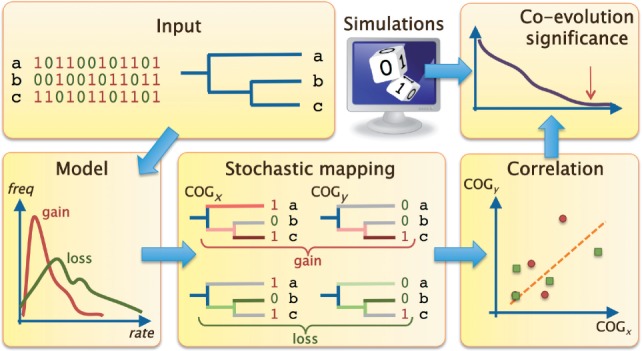
Methodology outline. Given an input of phyletic pattern and a phylogenetic tree, we detect correlated evolutionary histories and use simulations to infer significant co-evolving genes

Using a phyletic pattern of 4,593 COGs, which are present in at least one of the 282 prokaryotic genomes analyzed, we computed the network of co-evolving genes, in which two vertices (COGs) are connected only if they are significantly co-evolving (Fig. 1; also see Section 2 for details).

For 4,593 COGs there are 10,545,528 possible interactions. We only tested for co-evolution among 3,548 COGs with exchangeability above a predefined threshold (see Section 2), which resulted in 6,292,378 pairs.

### 3.2 Network properties

**Fig. 2.**
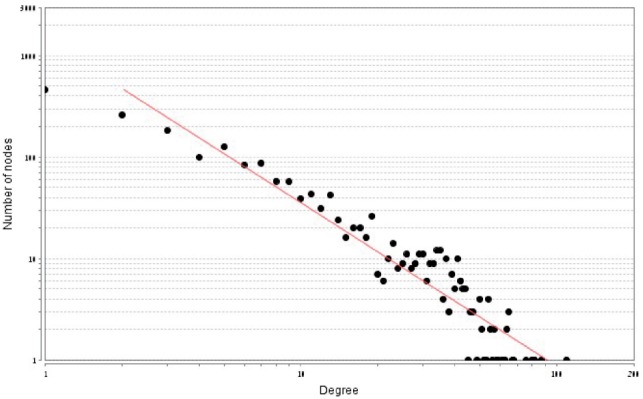
Degree distribution of the co-evolutionary network on a log–log scale. All 4,593 COGs are ranked according to their degree. In total, 1,940 COGs have at least one connection.

The network of co-evolving genes was found to be relatively highly connected with an average degree of 3.793. The degree varies substantially among genes in agreement with previous research (Cordero *et al*., 2008). Out of all COGs, 42.24% (1,940) were found to co-evolve with others (degree *>*0). The average degree (3.793) is substantially higher than the median degree (0) suggesting that the network of co-evolution relations does not fit a random network model. Moreover, a goodness-of-fit evaluation for a Poisson degree distribution was rejected with *P*-value *<*10E-100. In contrast, a linear regression between log the degree and log the number of nodes with the degree resulted with *R*^2^ = 0.919 (Fig. 2). Taken together, these results may suggest that a scale-free model is more suitable to describe the co-evolutionary network as compared with random models (Barabasi and Oltvai, 2004; Davids and Zhang, 2008). As illustrated in Supplementary Figure S2, the co-evolutionary network consists of 35 COGs with high number of co-evolutionary partners (degree ⩾50) and 536 COGs with multiple co-evolutionary partners (degree ⩾10). The Watts–Strogatz clustering coefficient (Watts and Strogatz, 1998) was found to be 0.526. This indicates relatively high transitivity in the co-evolutionary interactions.

### 3.3 Uncovering modular assemblies of genes

We have used the transitivity clustering algorithm (Wittkop *et al*., 2011) to cluster the network of co-evolution interactions into groups. We found 3,568 clusters, out of which 2,653 singleton clusters (COGs with no significant co-evolutionary partners), 326 clusters of two members, 101 clusters with three members, 42 clusters with four members, 13 clusters with five members and additional 41 clusters with at least six members up to the biggest cluster that consists of 30 COGs. Selected examples with at least six COGs included in the cluster and clear association with specific functions are depicted in Table 1 (See Supplementary File S3 for details).

**Table 1.** Clusters of co-evolving COGs associated with specific functions. The size corresponds to the number of COGs in the cluster. The suggested function is based on the annotation describing most of the cluster members as defined in the COG database

Cluster	Size	Suggested function
1	30	Flagellum and motility
5	13	NADH: ubiquinone oxidoreductase
11	9	Cobalamin (vitamin B12) synthesis pathway
13	8	Molybdopterin biosynthesis
14	7	UDP-N-acetyl processing
16	7	Hydrolysis of urea
17	7	F0F1-type ATP synthase
18	7	Type IV secretory pathway
22	7	Na-transporting NADH: ubiquinone oxidoreductase
31	6	Archaeal/vacuolar-type H -ATPase
33	6	Multisubunit Na+/H+ antiporter
35	6	Type III secretory pathway
36	6	Cobalamin (vitamin B12) synthesis pathway
38	6	Mu-like prophage
40	6	Flp pilus assembly

This co-evolutionary clustering reveals multiple assemblies of genes that have clear modular functional association (see Supplementary File S3 for the complete list of clusters). These members are both highly interconnected and relatively separated from all other clusters (i.e. few connections with genes outside the cluster), suggesting a shared functionality that has relatively low dependency on all other genes.

**Fig. 3.**
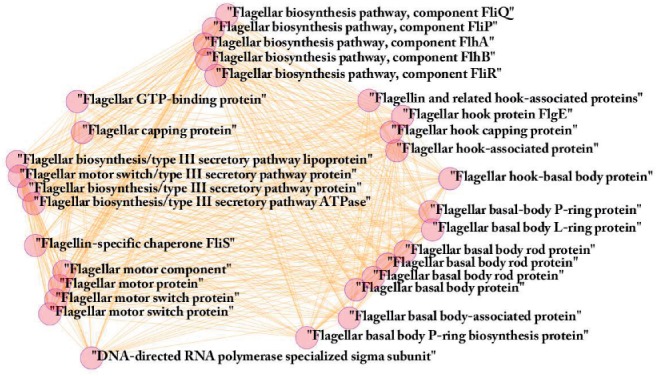
The flagellum-related cluster. This cluster contains 30 highly connected COGs (the nodes in the figure), all flagellar-related and is the biggest cluster of co-evolutionary genes

The largest cluster includes 30 COGs. All COGs belonging to the cluster are clearly related to flagellum functionality and motility (Fig. 3; see Supplementary File S3 for list of COGs and details). For all but one (COG1191), the COG description explicitly states its involvement in flagellum biology. A further inspection of COG1191: ‘DNA-directed RNA polymerase specialized sigma subunit’ revealed that it is also functionally associated with the flagellum. Several genes included in this COG encode for proteins that are related for the transcription regulation of the flagellar operon. For example, in *Escherichia coli*, it encodes the FliA protein: ‘RNA polymerase sigma factor for flagellar operon’.

We observed that in some cases, functional modules were separated into two clusters [e.g. the Cobalamin (vitamin B12) synthesis pathway in clusters number 11 and 36]. Moreover, some clusters do not present a clear functional modularity (Supplementary File S3). These results may reflect shortcoming of our methodology or gaps in the current functional annotations.

In some cases a cluster with clear function includes genes families that are described as ‘Uncharacterized’ or ‘Poorly characterize’ according to the COG annotation. We analyzed in detail two such cases: cluster number 38, related to ‘Mu-like prophage’ function and cluster number 18, related to the ‘Type IV secretory pathway’. Using these examples, we demonstrate the utility of the co-evolutionary network to predict functional annotations.

**Fig. 4.**
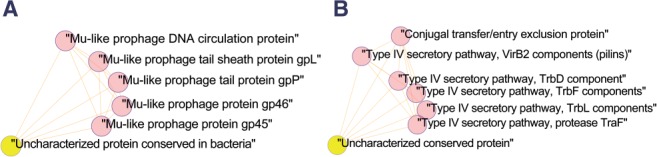
Functional modules of co-evolving genes that include an uncharacterized member. (**A**) ‘Mu-like prophage’ cluster (**B**) ‘Type IV secretory pathway’ cluster. Yellow nodes correspond to COGs that are uncharacterized

The ‘Mu-like prophage’ cluster is composed of the following seven COGs: COG3778, COG4228, COG4379, COG4381, COG4384 and COG4386 (Fig. 4A). For all except one of its members, the COG description explicitly states its association with ‘Mu-like prophage’ proteins. The COG description for COG3778 is ‘Uncharacterized protein conserved in bacteria’. Inspecting the annotation of genes within this COG reveals several examples which suggest that this member is also genuinely related to Mu-like propage proteins. For example, in *Haemophilus influenza* and *Vibrio cholerae*, these genes encode ‘Mu-like prophage FluMu protein gp48’.

Out of the seven COGs included in the Type IV secretory pathway cluster we found five COGs with COG descriptions that clearly depict them as components of the Type IV secretory pathway (COG5268, COG4959, COG3838, COG3846, COG3701) and an additional one described as “Conjugal transfer/entry exclusion protein” (COG5314), which fits the function of the entire cluster as all genes encode proteins that are part of the Type IV conjugation system. However, COG5489 is described as “Uncharacterized conserved protein”. As visualized in Figure 4B, this COG has co-evolutionary interactions with all other six members of the cluster, however further inspection of genes that are members of this COG did not yield clear association with Type IV conjugation function. Analyzing other cellular association between this COG to the other members of the clusters using STRING (Szklarczyk *et al*., 2011) revealed association with one of the cluster members COG4959, ‘Type IV secretory pathway, protease TraF’. The signals include medium and high confidence for fusion and genomic proximity, respectively. Inspection of several chromosomal cassettes that include this uncharacterized COG5489 further suggests possible involvement with the Type IV conjugation system. For example, in *Caulobacter* sp. K31 and as compared with other genomes by Integrated Microbial Genomes (IMG) chromosomal cassettes (Markowitz *et al*., 2012) multiple conjugation related proteins are found in the genome in tandem with COG5489. These neighboring proteins include COG2948, Conjugation TrbI family, COG4504, P-type conjugation transfer protein TrbG, COG3701, conjugation transfer protein, COG3846, P-type conjugation transfer protein TrbL, COG5314, P-type conjugation transfer protein TrbJ, COG3451, component of IV transporter system, COG5268, putative conjugal transfer TrbD transmembrane protein, COG3838, conjugal transfer TrbC and COG3962, P-type conjugative transfer ATPase TrbB (Supplementary Fig. S3). Finally, we used the amino acids alignment of COG5489 to search the PDB for predicted structural similarity using HHPred (Soding *et al*., 2005). We have found a significant predicted similarity (*E*-value *<* 3.7E-16) with PDB structure 1B12, A Signal peptidase. This may provide a clue towards elucidating the relevance of this gene to the Type IV conjugation system as previous studies had found plasmid-encoded signal peptidase genes that are related to conjugation (Chaston *et al.*, 2011). Taken together, these analyses demonstrate the potential of using co-evolutionary interactions for functional annotation.

### 3.4 The overlap of the co-evolutionary network with other cellular networks

We next tested the extent to which the co-evolutionary network overlaps other system-biology related networks that had been reported to be informative of functional association between proteins (see Section 2 for details regarding networks reconstruction). These include PPI (e.g. Juan *et al*., 2008), genomic proximity and gene-order (e.g. Dandekar *et al*., 1998; Overbeek *et al*., 1999), co-expression (e.g. Chen and Dokholyan, 2006), gene fusion (Enright *et al*., 1999; Marcotte *et al*., 1999; Yanai *et al*., 2001) and metabolic networks (e.g. Spirin *et al*., 2006). For the 4,593 COGs in this study, there are 10,545,528 possible connections. For a specific network type we measured the number of reliably reconstructed connections divided by the total number of possible connections (column ‘Frequency’ in Table 2). For a network *X* and the co-evolutionary network, we additionally computed the number of connections that are both in *X* and in the co-evolutionary network out of the total number of connection in the co-evolution network (column ‘Conditional frequency’in Table 2). The highest conditional frequency, 0.173, was computed for the genomic proximity network, indicating that 17.3% of the co-evolutionary connections are also significant genomic proximity connections. In other words, ~83% of the co-evolutionary edges are not predicted to be genome proximity edges with high confidence. When the genomic proximity network is reconstructed with lower requirement for proximity (low confidence of 150 instead of high confidence of 700 as defined by the STRING database, see Supplementary Table S2 for details), we still find that only ~36% of the co-evolutionary connections are between neighboring genes. It is clear that genomic proximity substantially increases the probability of co-acquisition and co-deletion of genes and hence it is expected that many co-evolutionary dependencies rise from proximity. However, these results suggest that a major part of all co-evolutionary dependencies may rise between genes that are not in a tight physical proximity.

**Table 2.** The frequency of interactions within various cellular networks and the conditional frequency of interaction with respect to co-evolutionary interactions.

A					B			
Network type	Frequency	Conditional frequency	Enrichment ratio	*P*-value	Frequency	Conditional frequency	Enrichment ratio	*P*-value
Genomic proximity	0.000374	0.173	461	≈0	0.000374	0.453	1210	0
Gene fusion	0.0000894	0.0186	208	≈0	0.0000894	0.062	693	0
Co-expression	0.00161	0.0606	37.7	≈0	0.00161	0.211	131	0
PPI	0.001	0.0248	24.7	3.2E-211	0.001	0.0972	96.9	9.02E-286
Metabolic	0.00585	0.159	27.11	≈0	0.00585	0.495	84.69	≈0

‘Frequency’ is the number of connections of that type divided by all possible pairs (10,545,524), ‘Conditional frequency’ in Table 2, part A is the number of connections within each network type that also co-evolve divided by the total number of co-evolving edges (8,710). In Table 2, part B, ‘Conditional frequency’ is this number of connections within each network type that are both co-evolving and functional information divided by all co-evolutionary connections that are also functionally informative (1,904). The reported *P*-value is computed against a null hypothesis that there is no enrichment (i.e. the enrichment ratio equals 1) as determined by Fisher's exact test.

For each network type, we further compute an enrichment ratio, which is the ratio of the conditional frequency and the frequency. Explicitly, for network type *X*, the enrichment value approximates the ratio: Pr(edge ? *X* | edge ? *C*) / Pr(edge ? *X*), where *C* is the set of all co-evolving edges. For example, for PPI, this value is an estimate for the increase in probability for an edge to be involved in PPI when it is known to co-evolve, over the baseline probability of an edge to be involved in PPI. As shown in Table 2, there is a very high and significant enrichment (461-fold) of the genomic proximity network. The lowest enrichment was observed with the PPI network, indicating that within the co-evolving edges, there is only 24.7 fold enrichment for PPI edges.

We define functionally informative edges to be edges that connect COGs that share the same functional annotation according to the eggNOG database annotation (Muller *et al.*, 2010). We repeated the analysis above, this time considering only edges that are both between co-evolving COGs and that are functionally informative. The results presented in Table 2 (part B) indicate that the enrichment ratio increases for all network types when considering only functionally informative edges. This suggests that considering only informative edges increases the agreement between the co-evolutionary signal and other system-biology measures. We observe that the highest conditional frequency is found for the genomic proximity network: 45.3% of functionally informative co-evolutionary connections are also inferred to be significant genomic proximity connections. Importantly, this result suggests that over half of the functionally informative co-evolutionary connections could not be directly inferred by mere physical proximity. For the PPI network the conditional frequency with functionally informative co-evolutionary connections is only 9.72%. This suggests that *>*90% of the functionally informative co-evolutionary connections are between genes that may not be in direct physical interaction (as determined by their relatively low PPI score). Similar results were obtained when the PPI interactions with lower confidence were considered (Supplementary Table S2 provides all the results given in Table 2 (part A) for low and medium confidence for the five network types).

## 4 DISCUSSION

We have reconstructed the co-evolutionary network among genes using a novel probabilistic methodology. We suggest that this approach balances well between two objectives. First, the requirement to analyze large datasets of hundreds of genomes, which is currently infeasible with explicit co-evolutionary models (e.g. Barker *et al*., 2007). Second, the requirement for accuracy and probabilistic inference of significant co-evolutionary interactions, which is lacking when using the maximum parsimony approach (e.g. Cordero *et al*., 2008).

Our results suggest that ~40% of all COGs co-evolve with at least one other member. Furthermore, the inferred co-evolutionary network is significantly enriched in the fraction of connections shared with other cellular network types. Indeed, in previous studies, high agreement between co-evolutionary signal and cellular interaction data was assumed, and hence the cellular interaction data were used as proxy for co-evolution signal (Cordero *et al*., 2008; Tuller *et al.*, 2010). Here, we took a different approach, in which the agreement between cellular interaction data and co-evolutionary signal was not assumed but rather, measured. Our data show that whereas agreement exists, it is far from perfect and the agreement also varies depending on the type of cellular interaction chosen. Although previous studies reported that interacting proteins tend to co-evolve (Juan *et al*., 2008), we show that it may be misleading to take PPI as proxy for co-evolution, since a small fraction of the co-evolutionary connections are between genes involved in PPI. We note that more research is needed to determine to what extent the lack of overlap between the different networks points to inaccuracies in methodologies and data or to inherent properties of the evolutionary and cellular networks. For example, it is clear the current PPI network is only partially known, especially in bacteria that are evolutionary divergent from model bacterial species such as *Escherichia coli*.

Whereas in prokaryotes, protein families typically contain one copy per species (Ranea *et al*., 2007), in eukaryotes, gene families often comprise hundreds of members. Thus, co-evolutionary signal may reside in gene families that are expanding and shrinking in a correlated manner. Our method only considers presence and absence of COGs, and would hence fail to detect such co-evolutionary pattern. This suggests that birth and death models for gene family sizes should be integrated in co-evolutionary detection method, as was done within the maximum parsimony framework (Cordero *et al*., 2008).

To summarize, we have developed a methodology to infer pairs of co-evolving genes. Using this methodology allows the reconstruction of a co-evolutionary network of COGs. Clustering of the co-evolutionary network reveals many examples of modular assemblies of genes that take part in a specific biological process. Our results suggest that co-evolutionary modules can be a valuable tool in genome annotation efforts.
